# The Association Between Different Levels of Suppressed Viral Load and the Risk of Sexual Transmission of HIV Among Serodiscordant Couples on Antiretroviral Therapy: A Systematic Review

**DOI:** 10.1007/s10461-025-04976-5

**Published:** 2025-12-17

**Authors:** Pascal Djiadeu, Housne Begum, Michelle Haavaldsrud, Giovanna Busa, Jeffery Dansoh, Phu Van Nguyen, Taline Ekmekjian, Annie Fleurant, Stephan Gadient, Chris Archibald

**Affiliations:** 1 Sexually Transmitted and Blood Borne Infections Surveillance Division, Centre for Communicable Diseases and Infection Control, Infectious Diseases and Vaccination Programs Branch, Public Health Agency of Canada, 130 Colonnade Rd., Ottawa, ON K1A 0K9 Canada; 2 PHAC Library, Public Health Agency of Canada, Ottawa, ON Canada

**Keywords:** Epidemiology, HIV and AIDS, Public health, Sexually transmitted diseases, Systematic review, Antiretrovirals, Viral load suppression, Serodiscordant couples, HIV transmission, < 1000 copies/mL

## Abstract

The risk of sexual transmission of HIV in serodiscordant couples when the partner living with HIV (PLHIV) uses antiretroviral therapy (ART) and maintains a suppressed viral load (VL) (< 200 copies/mL) is estimated at 0.00 transmissions per 100 person-years (95% CI 0.00–0.10). A recent review reported an almost zero transmission risk for VL < 1000 copies/mL but lacked quantitative risk estimates. This systematic review aimed to quantify transmission risk across VL levels. A literature search (November 2023, updated January 2025) identified 152 studies, with 9 included in the analysis. Seven possible transmissions occurred when the PLHIV had a VL of < 1000 copies/mL at the last measurement before the estimated time of transmission. However, VL at the exact time of transmission was unknown and VL > 1000 copies/mL cannot be excluded. This review suggests sexual transmission is very rare with recent VL < 1000 copies/mL however, future research is required to establish precise risk estimates.

PROSPERO Number: CRD42023476946.

**Supplementary Information:**

The online version contains supplementary material available at 10.1007/s10461-025-04976-5.

## Introduction

Human Immunodeficiency Virus (HIV) transmission remains a significant global public health concern. In 2023, approximately 40 million people were living with HIV, of whom 30.7 million (77%) were on treatment, and 1.3 million people were newly infected with HIV [1]. As a response to the goal to end HIV by 2030, the United Nations has set 2025 global [2] 95-95-95 targets in which 95% of people living with HIV (PLHIV) know their status, 95% of PLHIV who know their status are on treatment, and of those on treatment, 95% are virally suppressed [1]. In 2023, it was estimated that approximately 28.7 million PLHIV (72% of all PLHIV and 93% of PLHIV on treatment) were virally suppressed [2].

Antiretroviral therapy (ART) improves the lives of PLHIV and prevents their sexual partners from acquiring HIV since a suppressed viral load (VL) (< 200 copies/mL) prevents onward HIV transmission [3]. This concept is referred to as Undetectable = Untransmittable (U = U) [4] as a result of a health promotion campaign initiated in 2016 by the Prevention Access Campaign, a health equity initiative with the goal of ending the HIV/AIDS pandemic and associated HIV-related stigma. Based on the same evidence, the Treatment as Prevention (TasP) strategy recommends widespread access to ART to reduce population-level HIV transmission [5, 6].

In 2018, a systematic review and meta-analysis conducted by LeMessurier et al. [3] found that, according to criteria established by the Canadian AIDS Society [7], the risk of sexual transmission of HIV is negligible [7] when the PLHIV is on ART with a suppressed VL of less than 200 copies of HIV RNA/mL, as documented using regular testing every 4 to 6 months [3]. Djiadeu et al. [8] in 2023 reviewed five years of additional data and updated these findings to ascertain an estimated risk of sexual transmission of HIV of 0.00 transmissions per 100 person-years (95% CI 0.00–0.10) in this specific situation. For VL levels above 200 copies/mL in PLHIV on ART, LeMessurier et al. [3] found a small number of cases of HIV transmission and assessed the overall risk of sexual transmission of HIV as low [7]; however, the authors did not set an upper bound for VL levels in their review. Broyles et al. [9] conducted a systematic review in 2023 and concluded that the risk of HIV transmission through sexual contact is almost zero when the PLHIV is on ART and has a suppressed VL of less than 1000 copies/mL. However, the risk of sexual transmission of HIV was not quantified. Broyles et al. [9] did note two cases of HIV transmission with VL < 1000, but in both cases the VL measurement was done at least 50 days before the date of HIV transmission. In addition, Birungi et al. [10] reported 3 linked transmissions from PLHIV on ART with VL < 1000, although in all cases the most recent VL measurement was done up to 6 months before seroconversion.

Precise estimates of the risk of sexual transmission at sustained VL levels between 200 and 1000 copies/mL remain a significant gap in the literature. Addressing this gap by quantifying these risks would complement previous work on the transmission implications of low VL levels and reinforce the importance of VL testing as part of comprehensive HIV treatment and long-term care. The primary objective of this review was to quantify the risk of sexual transmission of HIV for different levels of VL (primarily in the range 200–1000 copies/mL) among serodiscordant couples where the PLHIV is on ART. By analyzing reported transmission cases within this VL range, the review highlights key limitations in the existing body of evidence and provides a foundation for future research. Unlike earlier reviews, this study applies structured assessments of bias and evidence certainty and uses SWIM methodology to clearly synthesize findings where a meta-analysis is not feasible.

## Methods

A comprehensive literature search was conducted on November 3, 2023, and updated on January 15, 2025, in the following databases: MEDLINE via Ovid, Embase via Ovid, the Cochrane Central Register of Controlled Trials (CENTRAL) via Ovid, Global Health via Ovid and Scopus. The search was limited to studies published after January 2000. The search strategy, developed collaboratively with a health information professional (TE), utilized both text and relevant index terms to locate studies addressing VL, ART, and HIV transmission among serodiscordant couples. The MEDLINE search strategy served as a baseline and was adapted as necessary for consistency across all databases. A detailed account of the search strategy can be found in Appendix 1 and eligibility criteria has been published elsewhere [11]. Key eligibility criteria included ART therapy, consistent VL testing (i.e., every 3 to 6 months) and a phylogenetic test confirming genetic linkage of HIV to the index partner. In designing this systematic review, neither patients nor members of the public were involved.

### Research Questions

What is the risk of sexual transmission of HIV in serodiscordant couples when the PLHIV is on ART and has a viral load between 200 and 1000 copies/mL?

### Meta-analysis

Although the team initially planned to conduct a meta-analysis to quantify the risk of HIV transmission at different VL levels, this was not feasible due to substantial heterogeneity in study characteristics and insufficient detail in reported findings, particularly regarding person-years at different VL levels. These limitations in the available data (see below) prevented the use of a standard meta-analysis combining results across several studies. While meta-analysis remains the gold standard for assessing the effects of interventions [12], the team was not able to quantify the risk of transmission in this synthesis and therefore only reports the number of HIV transmissions at different VL levels (< 200 copies/mL and between 200 and 1000 copies/mL). An individual participant data (IPD) meta-analysis was not conducted at this time but is being considered for future work.

## Literature Review and Study Selection Process

This review adhered to the Preferred Reporting Items for Systematic Review (PRISMA) [13] and Grading of Recommendations, Assessment, Development and Evaluation (GRADE) guidelines [14], and followed a published predefined study protocol [11]. As defined in the PRISMA 2020 checklist [13] (Appendices 2 and 3), a Synthesis Without Meta analysis (SWIM) guidance [12] was conducted (Appendix 4). The SWIM guideline, developed by Cochrane, facilitates clear reporting in reviews, offering alternative methods of synthesis where meta-analysis is impractical and promotes transparency in research methodologies.

All screening, data extraction, and quality assessment procedures were carried out independently and duplicated by three of the authors (GB, JD, PN). Discrepancies and disagreements were resolved through consensus among the authors, with unresolved issues settled by a fourth author (PD or HB). Articles meeting the inclusion criteria based on title and abstract screening underwent full-text review by two independent authors (GB, JD). Additionally, references of included studies were manually searched to identify any further relevant studies.

**Fig. 1:**
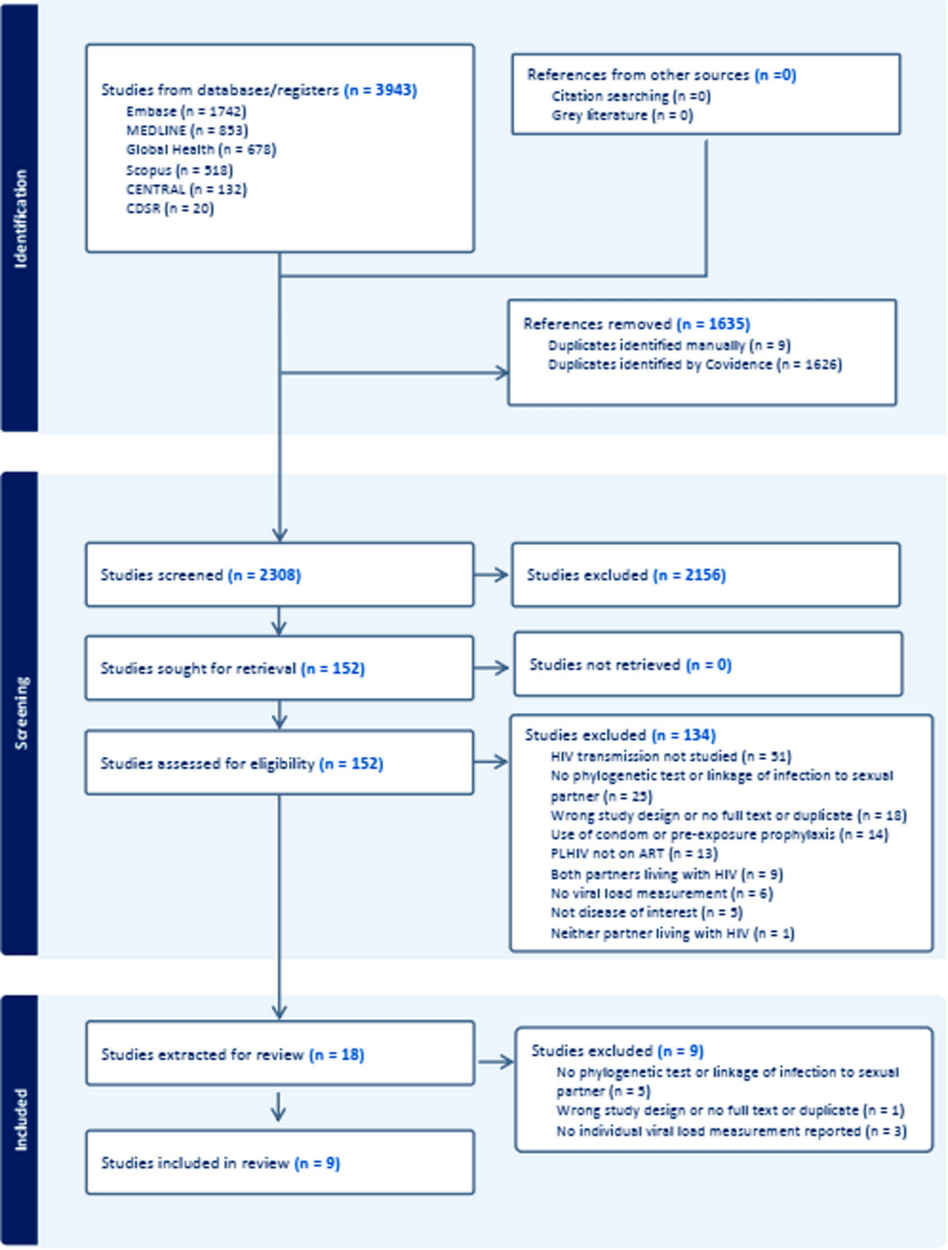
PRISMA flow diagram-Adapted from the PRISMA 2020 [13]

In cases where data or information were missing, attempts were made to contact the original authors. Finally, exclusions of publications were confirmed by a third author (PD, HB, CA or MH), along with reasons for their exclusion. A PRISMA flow chart adapted from the PRISMA 2020 (Fig. 1) shows the process of study selection.

### Data Extraction and Management

Following the full-text screening and study selection process, selected studies underwent data extraction, involving a comprehensive review of the full text to gather relevant information. The list of extracted variables is presented in the protocol [11]. Two independent authors utilized this form to extract data from the studies (GB and JD). After extraction, the authors' records were cross-checked, and any discrepancies were resolved through consultation with a third author (PD, HB, CA or MH).

### Risk of Bias Assessment

Prognostic studies were assessed using the Quality In Prognosis Studies (QUIPS) tool [15] (Appendix 5). Cochrane Prognosis Methods Group recommends the use of the QUIPS tool to assess risk of bias in prognostic factor studies. The QUIPS tool requires six important domains to be critically appraised when evaluating validity and bias in studies of prognostic factors: i) study participation, ii) study attrition, iii) prognostic factor measurement, iv) outcome measurement, v) study confounding, and vi) statistical analysis and reporting. Risk of bias was assessed as “critical risk”, “serious risk”, “moderate risk”, “low risk”, or “no information”. Publication bias was not assessed as this review did not include the minimum requirement of ten articles for each research question.

### Data Synthesis

Descriptive statistics from the studies included were extracted and summarized in tables. To aid comprehension of the risk of HIV transmission, data were combined by categorizing studies according to the VL levels of PLHIV, in accordance with SWIM guidelines [12]. Summary statistics are presented as a percentage for categorical variables. Due to the small number of studies, formal methods to assess studies’ heterogeneity, such as subgroup analysis and meta-regression, were not possible. Therefore, using an informal method, [12] we explain any heterogeneity by narratively describing the study characteristics and study design (Table 1). Given the small number of studies included subgroup analyses and sensitivity analyses were not required.

**Table 1:** Baseline characteristics of the 9 included studies

Author and study year	Study name	Country	Study type	Sample size	Types of couples	Phylogenetic testing
Donnell 2010 [16]	Partners in Prevention HSV/HIV Transmission Study	Botswana, Kenya, Rwanda, South Africa, Tanzania, Uganda, and Zambia	Prospective Cohort	3381 couples (3032 did not initiate ART and 349 initiated ART)	100% heterosexual	Yes
Birungi 2015 [10]	Highly Active Antiretroviral therapy and Prevention (HAARP) study	Uganda	Prospective Cohort	586 couples (249 ART at enrollment, 99 ART during study and 238 no ART)	100% heterosexual	Yes
Rodger 2016 [17]	PARTNER study	Austria, Belgium, Denmark, Finland, France, Germany, Ireland, Italy, Portugal, Spain, Sweden, Switzerland, The Netherlands, and UK	Prospective Cohort	888 couples	61.7% heterosexual 38.3% MSM	Yes
Mujugira 2016 [18]	PARTNER PrEP Study [19]	Kenya and Uganda	Authors used data from the placebo arm of PARTNER PrEP Randomized control trial	4747 couples (1584 treated with enofovir disoproxil fumarate [TDF]; 1579 to TDF coformulated with emtricitabine [TDF-FTC] and 1584 placebo arm; on ART but no-PrEP))	100% heterosexual	Yes
Eshleman 2017 [20]	HPTN 052	Africa, Asia, and the Americas	Randomized Control Trial	1763 couples	97% heterosexual 3% unspecified	Yes
Bavinton 2018 [21]	Opposites Attract	Australia, Brazil, and Thailand	Prospective Cohort	343 couples	100% MSM	Yes
Rodger 2019 [22]	PARTNER2 study	Austria, Belgium, Denmark, Finland, France, Germany, Ireland, Italy, Portugal, Spain, Sweden, Switzerland, The Netherlands, and UK	Prospective Cohort	782 couples	100% MSM	Yes
Sun 2020 [23]	–	China	Retrospective cohort	246 couples (104 treated with PrEP and 142 couples not treated with PrEP)	100% heterosexual	Yes
Han 2024 [24]	–	China	Randomized Control Trial	743 couples (372 couples in additional intervention group and 371 couples in control group)	100% heterosexual	Yes

^*^PrEP pre-exposure prophylaxis, *MSM* men who have sex with men

### Certainty of Evidence

GRADE was used to assess the certainty of evidence considering bias risk of the trial, consistency of effect, imprecision, indirectness, dose response, and residual confounding [14]. Some modifications to the GRADE tool were made to rate the certainty of evidence in the absence of a single estimate of effect [25].

## Results

The search yielded 3943 articles for screening, of which 1635 were identified as duplicates, leaving 2308 studies for title and abstract screening. Of these, 2026 articles were excluded, resulting in 151 studies for full text review. Ultimately, 18 articles were deemed viable, including 8 relevant systematic reviews selected for data extraction. No new studies were deemed eligible from within those systematic reviews. Nine studies were further excluded after data extraction of the 18 relevant studies (Appendix 7). Therefore, only 9 studies were eligible to be reported and included in the final review (Appendix 6). Figure 1 shows the process of identifying the potential articles and the reasons for article exclusion at each stage of screening. Appendices 6 and 7 describe the reasons for inclusion and exclusion of articles considered for extraction, respectively.

Table 1 shows a summary of the characteristics of the 9 studies included within this review. Three of the studies, Rodgers et al. [17], Rodgers et al. [22], and Bavinton et al. [21], were analysed for risk of bias and certainty of evidence in our previous review [8] (Table 2). Where applicable, we included the remaining 6 articles as new evidence. Overall, there were 6 observational studies (5 prospective [10, 16, 17, 21, 22] and 1 retrospective [23]) and 3 randomized control trials [18, 20, 24]. Each study focused on HIV transmission in serodiscordant couples where the PLHIV was on ART. Studies that report pre-exposure prophylaxis (PrEP) use are distinguished in Table 1.

**Table 2:** GRADE Summary of findings and certainty of evidence

Certainty assessment							No of possible HIV transmission cases^*^	Certainty of Evidence (GRADE)
Name of studies	Study design	Risk of bias	Inconsistency	Indirectness	Imprecision	Publication bias		
Outcome: Number of HIV transmissions for VL < 1000								
Pooled evidence of Bavinton et al. and Rodgers et al. found in Djiadeu et al. [8]	Cohort	Not serious^a^	Not serious	Not serious	Serious^b^	Undetected	0	⨁⨁⨁⨁ High
Sun et al. [23]	Cohort	Not serious	Not serious	Not serious^c^	Serious^d^	Not assessed^e^	0	⨁⨁⨁◯ Moderate^f^
Donnell et al. [16]	Cohort	Serious	Not Serious	Not Serious	Serious^g^	Not assessed^e^	0	⨁◯◯◯ Very low^h^
Eshleman et al. [20]	RCT	Not serious	Not Serious	Not serious	Serious^i^	Not assessed^e^	1	⨁⨁⨁◯ Moderate^j^
Mujugira et al. [18]	RCT	Serious	Not Serious	Not serious^k^	Serious^l^	Not assessed^e^	1	⨁⨁◯ ◯ Low
Birungi et al. [10]	Cohort	Serious	Not serious	Not serious	Serious^m^	Not assessed^c^	3	⨁◯◯◯ Very low
Han et al. [24]	RCT	Not serious	Not serious	Not serious	Serious^n^	Not assessed^c^	2	⨁⨁⨁◯ Moderate

*RCT* randomized control trial

The studies did not clearly report on the subpopulation of interest

**Fig. 2:**
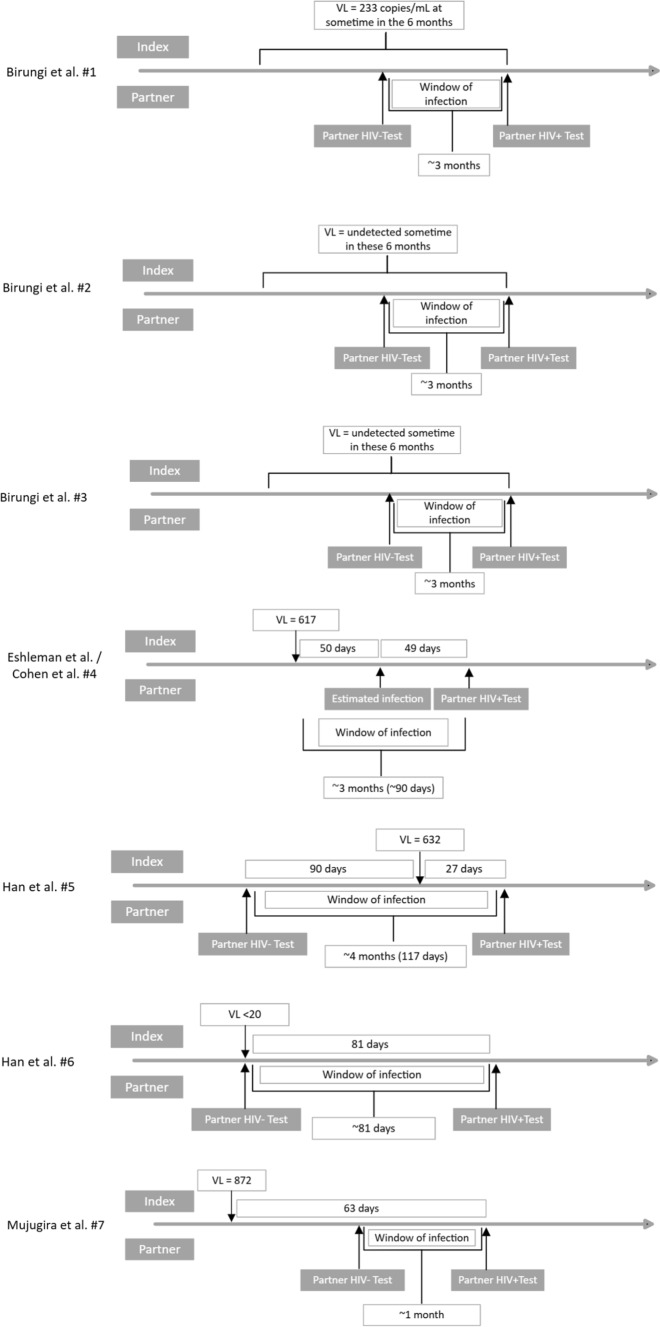
Timelines of possible cases who seroconverted when HIV positive partners were receiving ART and had a viral load of < 1,000 copies/mL and who were phylogenetic linked

^*^For further details, see text, Table 3 and Fig. 2

^a^Risk of bias was assessed as low for one study and as moderate for the other. However, both studies reported consistent results

^b^Imprecision: Total numbers do not meet the optimum sample size. Because sample size and follow-up time were insufficient (i.e., below 2,000 participants and 4,000 person-years), imprecision was rated as serious

^c^Sun et al., was rated −0.5 down for indirectness because the primary outcome was on PrEP during natural conception and not HIV transmission. Although, considering the secondary outcome was HIV transmission, we believe indirectness is not a serious threat

^d^Sun et al., was rated serious for imprecision due to small sample size. But considering the influence of the dose–response gradient, we have increased certainty in the effect regardless of imprecision

^e^Publication bias was not assessed as this review did not include the minimum requirement of 10 articles [26]

^f^The overall rating for Sun et al., is moderate. Dose response gradient: there was a dose–response relationship between the viral load and HIV transmissions, so rated up for a dose–response gradient [27]

^g^Imprecision was rated as serious, and the certainty of evidence was rated down by 1.5 due to confounding and small sample size

^h^The overall certainty of evidence was rated as very low

^i^Imprecision was rated as serious, and the certainty of evidence was rated down by 0.5 due to confounding and 0.5 due to a small sample size

^j^The overall certainty of evidence was rated as moderate

^k^Indirectness is not serious. Mujugira et al., was rated down 0.25 due to indirectness because the primary outcome of the study was not directly related to this SR’s outcome, but the secondary outcome was related

^l^The certainty of evidence was rated down −2 due to imprecision and risk of bias

^m^Imprecision was rated as serious, and the certainty of evidence was rated down by 1.5 due to confounding and small sample size

^n^The certainty of evidence was rated down 1 due to imprecision

The transmissions between the serodiscordant couples within each study were categorized by VL prior to the time of transmission, notably < 200 copies/mL and between 200–1000 copies/mL. Six studies specifically examined possible transmissions with VL levels under 200 copies/mL [10, 17, 21–24] and 5 studies examined possible transmissions with VL levels between 200 and 1000 copies/mL [10, 16, 18, 20, 24]. The studies examining VL < 1000 copies/mL reported 13 possible transmissions; however, only 7 cases (Table 3, Fig. 2) are considered for further analysis since the remaining 6 cases were excluded because the VL measurements reported did not correspond to the period prior to the most likely date of HIV transmission while the PLHIV was on ART (Table 4).

**Table 3:** Characteristics of possible cases who seroconverted when HIV positive partners were receiving ART and had a viral load of < 1,000 copies/mL and who were phylogenetic linked

Case #	Author	SC Age	SC Sex	Viral load(s) of HIV + partner (copies/mL) and dates of collection	Time between VL collection and first positive HIV test in seronegative partner	Likely date or time period of transmission based on analysis	Time between VL collection and likely date of transmission	ART duration prior to first positive HIV test in seronegative partner	ART regimen prior to first positive HIV test in seronegative partner	Rationale
1	Birungi et al. 2015 [10]	29	F	233 Sample collected up to 6 months before first positive HIV test	NA (VL was taken up to six months before the first positive HIV test) ^a^	Transmission may have occurred in the 3 months before first positive HIV test (seronegative partners tested q3 months)	VL was taken up to six months prior to transmission event ^a^	6 months	AZT, 3TC, EFV Self-reported adherent to therapy	Case provides some support of HIV transmission at VL < 1000 copies/mL
2	Birungi et al. 2015 [10]	47	M	Not detected Sample collected up to 6 months before first positive HIV test	NA (VL was taken up to six months before the first positive HIV test) ^a^	Transmission may have occurred in the 3 months before first positive HIV test (seronegative partners tested q3 months)	VL was taken up to six months prior to transmission event ^a^	9 months	AZT, 3TC, NVP Self-reported adherent to therapy	Case provides some support of HIV transmission at VL < 1000 copies/mL
3	Birungi et al. 2015 [10]	45	F	Not detected Sample collected up to 6 months before first positive HIV test	NA (VL was taken up to six months before the first positive HIV test) ^a^	Transmission may have occurred in the 3 months before first positive HIV test (seronegative partners tested q3 months)	VL was taken up to six months prior to transmission event ^a^	65 months	AZT, 3TC, NVP Self-report adherent to therapy	Case provides some support of HIV transmission at VL < 1000 copies/mL
4	Extra Eshleman et al. 2017 [20]—case F cited in Cohen 2016 [28] (Included in Broyles et al. 2023 [9])	22	NA	Last VL before estimated infection date: 617 (at 50 days before infection)	50 days before estimated infection date and 99 days before first positive HIV test (Cohen et al. [28])	Estimated to be 1667 days after ART initiation (Cohen et al. [28])	VL was collected 50 days before estimated date of transmission (range 0–99 days)	1716 days	Treatment failure on their initial regimen Receiving second line ART (Broyles et al. [9]) No drug resistance	Case provides some support of HIV transmission at VL < 1000 copies/mL Eshleman et al. [20] excluded this couple and classified them as viremic (not reaching < 400 copies/mL) Cohen et al. [28] classified case as ART failure Broyles et al. [9] explained that “the VL test was done at least 50 days before transmission occurrence. This fact, coupled with inherent variability in molecular VL results, complicates interpretation of the events”
5	Han et al. 2024 [24] - Subject 1	41	M	Enrollment: NA 90 days after last negative HIV test and 27 days before first positive HIV test in partner: 632	27 days	Transmission may have occurred within the 117 days from last HIV negative test to HIV positive test	VL was collected 27 days before first positive HIV test however transmission could have occurred within the 117 days prior to first positive HIV test	110 days	NA	seroconverted within 110 days of initiating ART, aligning with previous findings that within 6 months represents the highest risk for serodiscordant couples starting ART
6	Han et al. 2024 [24] - Subject 3	53	F	Enrollment: NA At time of last negative HIV test and 81 days before first positive HIV test in partner: < 20	81 days	Transmission may have occurred within the 81 days from last HIV negative test to first HIV positive test	VL was collected on same day as last negative test and 81 days before first positive test in partner; transmission likely occurred within this 81-day period	159 days	NA	seroconverted within 159 days of initiating ART, aligning with previous findings that within 6 months represents the highest risk for serodiscordant couples starting ART
7	Mujugira et al. 2016 [18]—couple 3 (Included in Broyles et al. 2023 [9])	32	M	Enrollment: 694 Pre-ART: 824 86 days after ART: 872 > 6 months: 264	63 days (149 days (time from reported date of ART initiation to first positive HIV test) minus 86 days (time from reported date of ART initiation to HIV + partner VL test))	Transmission may have occurred within the one-month period before 149 days after ART initiation (days 119–149) Seronegative partner was tested monthly	Range of 33–63 days before estimated date of transmission (since seronegative partner was tested every month)	149 days (almost 5 months)	D4T, 3TC, NVP Pre-ART: 824 ≤ 6 months: 872 suggests suboptimal ART	This case does support HIV transmission at index VL < 1000 copies/mL Broyles et al. [9] explained “this transmission could have occurred when the VL was less than 1000 copies/mL; however, the potential absence of ART exposure and the 53-day gap between seroconversion and time of VL quantification makes it difficult to determine the true dynamics of this event.”

^a^Time between VL collection and first positive HIV test was not further specified

**Table 4:** Characteristics of excluded cases who seroconverted when HIV positive partners were receiving ART and had a viral load of less than 1,000 copies/mL and who were phylogenetic linked

Case #	Author	SC Age	SC Sex	Viral load(s) of HIV + partner (copies/mL) and dates of collection	Time between VL collection and first positive HIV test in seronegative partner	Likely date or time period of transmission based on analysis	Time between VL collection and likely date of transmission	ART duration prior to first positive HIV test in seronegative partner	ART regimen prior to first positive HIV test in seronegative partner	Rationale
8	Donnell et al. 2010 [16]	NA	M	6 month visit and before ART: ~ 52,481 copies/mL 12 month visit (3 months and 18 days after ART initiation): < 240 copies/mL	There are no VL measurements before first positive HIV test while the index case was on ART (the 6-month VL sample was collected pre-ART 6 months before first positive HIV test)	Transmission occurred in the 3-month window between the 9 and 12 month visits (seronegative partners tested q3 months)	There are no VL measurements before transmission while the index case was on ART	108 days (18 + 90 days). (ART initiated 18 days prior to 9 month visit and first positive HIV test was at 12 month visit)	Individual regimen not reported	There is no VL measurement before seroconversion with the HIV + partner being on ART VL quantification at seroconversion is unknown. It varied between ~ 52,481 and < 240 copies/mL
9	Eshleman et al. [20] – case A	52	NA	VL 38 days before ART start: 278,398 VL 30 days after ART start: Undetectable (< 400 copies/mL)	5 days but this VL measurement was after the most likely date of the transmission event	Estimated to be within the range of 18 days before ART start to a maximum of 13 days after Beast: 5 days before ART (−18, + 10) Poisson Fitter: 1 day after ART (−10, + 13) Fiebig stage: 42 days before ART to 12 days after	There are no VL measurements before the likely date of transmission while the index case was on ART	35 days	Initial ART regimen EFV/3TC/ZDV	Genetic analysis showed that seroconversion most likely occurred sometime during the period between 5 days before ART and 1 day after ART VL quantification at seroconversion is unknown. It varied between 278,398 and < 400 copies/mL
10	Eshleman et al. [20] – case B	24	NA	VL one day before ART start 87,202 VL 57 days before first positive HIV test: Undetectable (< 400 copies/mL)	57 days but this VL measurement was after the most likely date of the transmission event	Estimated to be within the range of 32 days before ART start to a maximum of 29 days after Beast: on day 0 of ART (−32, + 19) Poisson Fitter: not performed due to complex VL Fiebig stage: 8 days before ART to 29 days after	There are no VL measurements before the likely date of transmission while the index case was on ART	84 days	Initial ART regimen EFV/3TC/ZDV	Genetic analysis showed that seroconversion most likely occurred on day 0, the same day as ART start. The VL was very high before ART start (87,202 copies/mL) VL quantification at seroconversion is unknown. It varied between 87,202 and < 400 copies/mL
11	Eshleman et al. [20] – case C	54	NA	VL on day of ART start: 48,316 VL 28 days after ART start: Undetectable (< 400 copies/mL)	31 days but this VL measurement was after the most likely date of the transmission event	Estimated to be within the range of 7 days before ART start to a maximum of 36 days after Beast: 5 days after ART (−4, + 22) Poisson Fitter: 17 days after ART (0, + 34) Fiebig stage: 7 days before ART to 36 days after	There are no VL measurements before the likely date of transmission while the index case was on ART	59 days	Initial ART regimen EFV/3TC/ZDV	Genetic analysis showed that seroconversion most likely occurred sometime during the period between 5 days after ART start and 17 days after ART start VL quantification at seroconversion is unknown. It varied between 48,316 and < 400 copies/mL
12	Mujugira et al. 2016 [18]—couple 1	40	M	Enrollment: 332,514 Pre-ART: 1,434,082 84 days after ART: 738 > 6 months: < 80	There are no VL measurements before the first positive HIV test while the index case was on ART	Transmission may have occurred sometime within the one-month period before 56 days after ART initiation (days 26–56) Seronegative partner was tested monthly	There are no VL measurements before transmission while the index case was on ART	56 days	AZT, 3TC, NVP	Partner’s first positive HIV test occurred 56 days after ART start. The VL pre-ART was very high (1,434,082 copies/mL) VL quantification at seroconversion is unknown. It varied between 1,434,082 and 738 copies/mL
13	Mujugira et al. 2016 [18]—couple 2	53	M	Enrollment: 20,188 Pre-ART: 56,168 28 days after ART: 404 > 6 months: 160	There are no VL measurements before the first positive HIV test while the index case was on ART	Transmission may have occurred sometime within the one month period before the day of ART initiation (days −30 to 0) Seronegative partner was tested monthly	There are no VL measurements before transmission while the index case was on ART	0 days (same day)	TDF, 3TC, NVP	Partner’s first positive HIV test occurred on the same day as ART initiation. The pre-ART VL was very high (56,168 copies/mL) VL quantification at seroconversion is unknown. It varied between 20,188 and 56,168 copies/mL

^a^Beast analysis estimates the time since HIV infection using phylogenetic methods for analysis of sequence data (Eshleman et al. 2017)

^b^Poisson Fitter analysis estimates the time since HIV infection based on the accumulation of neutral mutation in viral population. (Eshleman et al. 2017)

^c^Fiebig stage is determined by 7 days before the partner’s last test where HIV RNA was undetectable to the date of the partner’s first HIV positive visit.

### Studies Examining Possible Transmissions for Viral Load < 200 Copies/mL

Bavinton et al. [21] and Rodger et al. [17, 22] reported no HIV transmissions for VL < 200 and these articles were examined by Djiadeu et al. [8] and others (Le Messurier et al. [3], Broyles et al. [9]). Djiadeu et al. [8] pooled data from Bavinton et al. [21] and Rodger et al. [22] to estimate an incidence of zero transmissions/100 PY (95% CI: 0.00–0.11). The risk of bias (RoB) was evaluated as moderate for the article by Bavinton et al., and low for Rodger et al. and the certainty of evidence for both studies was evaluated as high (Table 2). Our literature search did not find any new study with a high certainty of evidence.

A new study by Sun et al. [23] was found that analyzed transmissions in serodiscordant couples wanting to conceive where the HIV-positive partner had achieved viral suppression (< 50 copies/mL) for more than 6 months. This retrospective cohort study investigated 246 serodiscordant couples classified in two arms based on the intervention provided: 104 seronegative partners chose to receive PrEP, and 142 seronegative partners chose no prophylaxis. In the non-PrEP arm, the researchers found zero transmissions when participants had VL below 50 copies/mL, and notably, no transmissions occurred in either group during the study period. The overall risk of bias was rated as low, and the certainty of evidence was rated as moderate (Table 2). The overall certainty of evidence was downgraded due to confounding and a relatively small sample size of 142 serodiscordant couples that is lower than the optimal sample size of 400 [29].

Two cases from Birungi et al. [10] were reported with VL levels < 200 copies/mL before the estimated HIV transmission date. The overall risk of bias for this study was rated as moderate, and the certainty of evidence was rated as very low (Table 2). Note that in this study, blood for VL measurements was collected every 6 months, resulting in a long interval between the VL measurement date and the date of potential HIV transmission. More detailed information on these cases is provided below and in Table 3 and Fig. 2.

- In Case #2 of Birungi et al. [10], the PLHIV had a ‘not detected’ VL that was collected up to 6 months before case #2’s first positive HIV test (time not further specified). The PLHIV self-reported being adherent to ART and had been continuously on therapy at least 9 months before the partner’s first positive HIV test, but no historical VL data are reported except for the single measurement noted above. Since seronegative partners were tested every 3 months, the transmission event may have occurred in the 3 months before the first positive HIV test.
- In Birungi et al. [10] case #3, the PLHIV reported adhering to ART during the 65 months prior to the first positive HIV test. The previous VL was ‘not detected’ and was collected up to 6 months before the partner’s first positive HIV test (time not specified); this is the only VL value reported for this case. Transmission may have occurred in the 3 months before the first positive HIV test as seronegative partners were tested every 3 months.

Han et al. [24] reported a case of sexual transmission of HIV when the VL was < 200 copies/mL before the transmission date. The overall risk of bias for this study was rated as low risk and the certainty of evidence was rated as moderate (Table 2). In this case (subject 3 in Han et al.), the seronegative partner first tested HIV positive 159 days after the positive HIV partner-initiated ART. The most recent previous HIV negative test for subject 3 was 81 days before this seroconversion, on the same day that the HIV positive partner had a VL measurement of < 20 (no other VL data were reported). Further information on this case is provided in Table 3 and Fig. 2.

### Studies Examining Possible Transmissions for Viral Load 200–1000 Copies/mL

Five studies [10, 16, 18, 20, 24] were identified that reported cases of HIV transmission among serodiscordant couples where the HIV-positive partner had a VL between 200 and 1000 copies/mL before the transmission event. The overall certainty of evidence varied with assessments ranging from very low to moderate according to GRADE criteria (Table 2). These five studies reported 4 cases of HIV transmission where the VL before the estimated date of transmission was between 200 and 1000 copies/mL; these cases are described below and in Table 3 and Fig. 2.

- In Birungi et al. [10], case #1’s PLHIV self-reported being adherent to ART at the time of their partner’s first positive HIV test and had been on therapy for at least 6 months prior. The only VL measurement reported before the date of the first positive HIV test was 233 copies/mL, and this was collected up to 6 months prior to the first positive HIV test. In this study, seronegative partners were tested every 3 months, therefore transmission may have occurred in the 3 months preceding the first positive HIV test.
- In Eshleman et al. [20], a case described in the supplementary data (also referred to as case F by Cohen et al. [28]) reported a VL of 617 copies/mL in the PLHIV at the time of the last negative HIV test in the partner (50 days before the estimated infection date and 99 days before the first positive HIV test). The PLHIV had been on ART for 1716 days before the first HIV positive test. This PLHIV achieved viral suppression (VL < 400) within 6 months of starting ART, had treatment failure on their initial regimen after 441 days of ART and was switched to second line ART. Then the VL levels declined for the next 2½ years, spiked to > 1000 copies/mL about 2 months before the last negative HIV test, and the next VL measurement done was 617 copies/mL at the time of the last negative HIV test [20]. VL testing was conducted every three months [28].
- Han et al.’s subject 1 first tested HIV positive 110 days after the positive HIV partner-initiated ART. The most recent previous HIV negative test for subject 1 was 117 days before this seroconversion, and the HIV positive partner had a VL measurement of 632 copies/mL 27 days before this seroconversion date (no other VL data were reported).
- In Mujugira et al. [18], the seroconverted case in couple #3 tested positive for HIV 149 days after the PLHIV initiated ART. Given that seronegative partners were tested monthly, transmission may have occurred during the one-month period preceding day 149 (i.e., between days 119 and 149 after ART initiation). The VL of the PLHIV were reported as 824 copies/mL before ART start and 872 copies/mL at 86 days after ART start, or 33–63 days before the estimated transmission event.

## Discussion

The objective of this review was to further explore and quantify the risk of sexual transmission of HIV for different levels of VL among serodiscordant couples. This systematic review included 6 articles [10, 16, 18, 20, 23, 24] not included in the team’s previous review published in 2023 [8]. The results of the studies were stratified by the HIV positive partners’ VL measurement taken most proximal in time before the first positive HIV test of the seronegative partner or the estimated HIV transmission date.

Many studies [3, 18, 22, 28] have indicated that a PLHIV who is on ART and maintains viral suppression at < 200 copies/mL cannot sexually transmit the virus to others, as referred to as U = U [4]. Previously assessed studies included in two past reviews [3, 8] showed no transmissions when VL levels were < 200 copies/mL with a high certainty of evidence. Sun et al. [23], with moderate certainty of evidence, found no transmissions during natural conception among 142 serodiscordant couples when the HIV-positive partner consistently had VL < 50 copies/mL, which reinforces previous findings of no transmissions of HIV when VL are < 200 copies/mL (Table 2). Our current review found 3 reported cases where the VL prior to transmission was < 200 copies/mL in studies from Birungi et al. [10] (very low certainty of evidence) and Han et al. [24] (moderate certainty of evidence). However, in both cases from Birungi et al. the PLHIV’s VL was collected up to 6 months before the seronegative partner’s first positive HIV test and in the case from Han et al., the VL was collected 81 days before the first positive HIV test (Table 3, Fig. 2). Since seronegative partners were tested every 3 months in the Birungi et al. study, transmission in these cases could have occurred in the 3 months before the first positive HIV test. Consequently, the time interval between VL collection and possible date of transmission could be up to 3–6 months. Similarly, in the Han et al. study, there was a period of 81 days between VL measurement, and the first positive HIV test and infection likely occurred during this 81-day period. Given these long time-intervals, it is possible that the VL could have been higher than 200 copies at the time of the seroconversion and so it cannot be definitely concluded that these cases represent transmissions at VL < 200 copies/mL.

Our review found four possible transmission events when the VL was between 200 and 1000 copies/mL (Table 3, Fig. 2). In all these cases, there was a substantial time interval between VL collection and the possible date of transmission, ranging from approximately one (Mujugira et al. [18]) to six (Birungi et al. [10]) months. Note for Han et al. subject 1, VL may have even been collected after the date of transmission. Broyles et al. [9] cited two of these cases (Eshleman et al. [20], Mujugira et al. [18]) and explained that these transmissions could have occurred when the VL was less than 1000 copies/mL and noted that the extended time intervals between dates of seroconversion and VL quantification made it difficult to determine the true dynamics of these events. These four possible transmission events do not provide conclusive evidence that sexual transmission of HIV can occur when the HIV positive partner’s most recent VL was < 1000 copies/mL. The HIV positive partners in these cases had a VL measurement that varied between 33 days to 6 months before the possible date of transmission (and in one case, the VL may have been taken after transmission). Without more granular data on VL throughout the study period, and given the extended time intervals noted above, it is not possible to exclude the possibility that the VL measures were higher than 1000 copies/mL at the time of HIV transmission. In addition to the timing of VL measurement, discrepancies between reported and actual ART adherence may also contribute to uncertainty in interpreting transmission events. Most studies relied on self-reported adherence, which may not accurately capture missed doses or inconsistent use. Another possible explanation for this uncertainty is intermittent viremia, also referred to as viral blips. These are temporary increases in VL that can occur in individuals on ART, even when their overall viral suppression is maintained [30]. While typically brief and not a sign of treatment failure, viral blips may still pose a transmission risk if they occur around the time of sexual contact [30, 31]. According to the literature, viral blips can last from 3 days to an estimated 20–30 days, with amplitudes ranging from 50 to 950 copies/mL [32, 33]. This suggests that viral blips may occasionally be high and prolonged enough to facilitate transmission.

In 2023, the World Health Organization (WHO) [9] conducted a systematic review and stratified the risk to the seronegative partner by the VL of the PLHIV who was virally suppressed on ART, by less than 600 copies/mL, 600–1000 copies/mL, and above 1000 copies/mL. The WHO examined possible transmissions to seronegative partners at these varying VL when the PLHIV is on ART and found no definitive evidence of transmission when VL are < 600 copies/mL and very rarely when VLs are between 600 to 1000 copies/mL [9]. Another systematic review by Supervie and colleagues [34] found one HIV transmission from 1672 HIV-serodiscordant couples where the index partner had been on ART for at least 6 months. The authors suggested that estimating a confidence interval for the risk of HIV transmission may be unfeasible due to insufficient data to determine whether the reported transmission occurred before or after 6 months of ART when the PLHIV would have been stably suppressed [34].

This review highlights a key gap in the literature, the lack of precise estimates of HIV transmission risk at VL levels between 200 and 1000 copies/mL. Limitations in existing studies, such as infrequent VL monitoring and reliance on self-reported adherence, make it difficult to draw firm conclusions. More rigorous research with detailed VL data, objective adherence measures, and individual level transmission outcomes is needed to inform clinical guidance and public health messaging.

### Strengths

This systematic review adds to the growing literature exploring sexual transmission of HIV in serodiscordant couples when the PLHIV is taking ART and is virally suppressed. We performed a rigorous review of the literature including six studies not covered in previous reviews and clearly reported available data with thorough and transparent assessments of bias and certainty of evidence. We followed the SWIM guidelines which enabled clear narrative reporting in reviews in which alternative methods of synthesizing are used when a meta-analysis is not possible. Our review included nine studies, six observational and three randomized controlled trials. Transmission events were classified by VL prior to transmission, with seven possible cases examined in detail. Studies with high certainty of evidence consistently reported zero transmissions when VL was < 200 copies/mL. However, the examined cases with VL < 200 or between 200 and 1000 copies/mL were compromised by long intervals between VL measurement and transmission, and by low to moderate certainty of evidence, making it difficult to draw firm conclusions. By including detailed case level data and evaluating risk of bias and certainty of evidence using GRADE criteria, our review highlights a critical gap in the literature.

### Limitations

Originally a meta-analysis was to be performed as it is considered the gold standard of reviews. The proposed meta-analysis was to be conducted using raw data from individual participants, an approach which offers advantages such as a greater quantity of data, more flexibility in analytic methods, the ability to conduct subgroup analyses, and improved ability to detect and address biases [35]. However, a meta-analysis was not possible due to insufficient details reported and study heterogeneity. As a result, a precise estimate of the risk of sexual transmission of HIV at sustained VL levels between 200 and 1000 copies/mL in serodiscordant couples was not ascertained and remains a significant gap in the literature. In addition, this systematic review yielded a small number of eligible studies, and the studies included were found to have some imprecision and indirectness.

### Implications for Policy and Further Research

This review noted 7 possible sexual transmission events of HIV in the context of a PLHIV having a VL < 1000 copies/mL. However, it is not possible to correctly attribute these cases given the long-time intervals between the VL collection date and the likely transmission date. These findings highlight that sexual transmission of HIV in such situations occurs very rarely, if at all, as has been noted by other authors [9]. More research is needed to quantify the risk of sexual transmission of HIV between 200 and 1000 copies/mL and to clarify the dose–response relationship between risk of transmission and VL. Increasing the frequency of VL testing during studies would allow researchers to better ascertain the VL at the time the seronegative partner acquired HIV. We acknowledge, however, that it may not be feasible due to logistical considerations and the requirement for a very large sample size given the very low number of likely transmission events, as has been recognized by Broyles et al. [9] and others.

## Conclusions

This updated review supports earlier findings that there remains no evidence of HIV transmission to sexual partners when a PLHIV is on ART and maintains a suppressed VL of < 200 copies/mL. In addition, this review found that sexual transmission events of HIV appear to be very rare when the PLHIV has a VL < 1000 copies/mL. Of note, the studies reviewed had significant limitations making it impossible to conclude definitely that the cases represented transmission events below < 1000 copies/mL. Other significant limitations of this systematic review included the methodology, small sample size, and low-to-moderate certainty of evidence. Future research is required to further establish precise risk estimates of sexual HIV transmission in serodiscordant couples with VL between 200 and 1000 copies/mL. Such information would improve the understanding of the risk of sexual HIV transmission in the context of ART. It would also support the broader goal of improving screening and linkage to care to ensure that the benefits of ART and VL testing are accessible both for individual health and for the public health benefit of preventing onward transmission.

## Supplementary Information

Below is the link to the electronic supplementary material.

Supplementary file1 (DOCX 45 kb)

## Data Availability

Data extracted from the included studies will be presented in the Results and Appendices; however, full extraction data tables will be available upon reasonable request from the corresponding author.

## Abbreviations

AIDS: Acquired immunodeficiency syndrome
ART: Antiretroviral therapy
gbMSM: Gay, bisexual and other men-who-have-sex-with-men
GRADE: Grading of Recommendations, Assessment, Development, and Evaluation
HIV: Human immunodeficiency virus
IPD: Individual participant data
MSM: Men who have sex with men
PHAC: Public Health Agency of Canada
PLHIV: People/partner living with HIV
PREP: Pre-exposure prophylaxis
PRISMA-P: Preferred Reporting Items for Systematic review and Meta-Analysis Protocol
QUIPS: Quality in Prognostic Studies
RCT: Randomized controlled trials
SWiM: Synthesis Without Meta-analysis
SD: Standard deviation
STI: Sexually Transmissible Infection
U = U: Undetectable equals Untransmittable
WHO: World Health Organization.
