# Supplementary material for: The Association Between Different Levels of Suppressed Viral Load and the Risk of Sexual Transmission of HIV Among Serodiscordant Couples on Antiretroviral Therapy: A Systematic Review
Source: AIDS Behav. 2025 Dec 17;30(5):1538–53. doi: 10.1007/s10461-025-04976-5 (PMC13167889; doi:10.1007/s10461-025-04976-5)
Supplement: Supplementary file 1 — Supplementary file1 (DOCX 45 kb) [file 10461_2025_4976_MOESM1_ESM.docx]

**Appendix**

**Appendix 1: Search strategies**

**Summary of results**

| Database Searched | Segment | Dates searched | Additional results |
| --- | --- | --- | --- |
| Medline [Ovid] | 1946 to January 14, 2025 | 01/11/2023 – 15/01/2025 | 789 |
| Embase [Ovid] | 1974 to 2025 January 14 | 01/11/2023 – 15/01/2025 | 1664 |
| Scopus [Elsevier] | Inception-today | 2023-2025 | 467 |
| EBM Reviews – CENTRAL [Ovid] | December 2024 | 2023-2025 | 20 |
| Global Health [Ovid] | 1973 to 2025 Week 03 | 2023-2025 | 617 |

**Medline [Ovid]**

| Ovid MEDLINE(R) ALL <1946 to January 14, 2025> | | |
| --- | --- | --- |
| 1 | exp Anti-Retroviral Agents/ | 90762 |
| 2 | ((antiretroviral or anti-retroviral) adj (agent? or therapy)).tw,kf. | 58674 |
| 3 | (anti-hiv adj2 (agent? or drug? or therapy)).tw,kf. | 3881 |
| 4 | ((CCR5 adj3 (Receptor or Antagonist? or blocker?)) or Atazanavir-Sulfate or Darunavir or Indinavir or Lopinavir or Nelfinavir or Ritonavir or Saquinavir or Raltegravir Potassium or Maraviroc or Enfuvirtide or Cobicistat or Delavirdine or Didanosine or Emtricitabine or Lamivudine or Nevirapine or Rilpivirine or Stavudine or Tenofovir or Trichosanthin or Zalcitabine or Zidovudine).tw,kf. | 42982 |
| 5 | 1 or 2 or 3 or 4 | 133395 |
| 6 | Viral Load/ | 40638 |
| 7 | ((viral or virus) adj2 (load or burden or titer or titre)).tw,kf. | 47803 |
| 8 | ("copies/ml" or "cells/ml" or "cells per cubic millimeter" or "<=1000copies/mL").tw,kf. | 24831 |
| 9 | ((cells or copies) adj3 (millimeter or mm or ml or milliliter)).tw,kf. | 44965 |
| 10 | (undetectable or (limit? adj2 detect*) or "copies/*").tw,kf. | 211028 |
| 11 | or/6-10 | 305323 |
| 12 | 5 and 11 | 27980 |
| 13 | sexual behavior/ or unsafe sex/ | 73596 |
| 14 | Sexual Partners/ | 21385 |
| 15 | ((sex or sexual) adj3 (behavio?r* or unsafe* or partner* or transmi* or condomless)).tw,kf. | 68451 |
| 16 | ((seropositiv* or seronegativ* or seroconver* or serodiscordan*) adj4 (partner* or couple*)).tw,kf. | 1498 |
| 17 | 13 or 14 or 15 or 16 | 123102 |
| 18 | 12 and 17 | 847 |
| 19 | (202311* or 2024* or 2025*).dt,ez,ed. | 2013672 |
| 20 | 18 and 19 | 41 |

**Embase [Ovid]**

| Embase <1974 to 2025 January 14> | | |
| --- | --- | --- |
| 1 | exp antiretrovirus agent/ | 240518 |
| 2 | ((antiretrovir* or anti-retrovir*) adj (agent? or therapy)).tw,kf,dq. | 77064 |
| 3 | (anti-hiv adj2 (agent? or drug? or therapy)).tw,kf,dq. | 4891 |
| 4 | ((CCR5 adj3 (Receptor or Antagonist? or blocker?)) or Atazanavir-Sulfate or Darunavir or Indinavir or Lopinavir or Nelfinavir or Ritonavir or Saquinavir or Raltegravir Potassium or Maraviroc or Enfuvirtide or Cobicistat or Delavirdine or Didanosine or Emtricitabine or Lamivudine or Nevirapine or Rilpivirine or Stavudine or Tenofovir or Trichosanthin or Zalcitabine or Zidovudine).tw,kf,dq. | 63659 |
| 5 | or/1-4 | 277456 |
| 6 | virus load/ | 118178 |
| 7 | ((viral or virus) adj2 (load or burden or titer or titre)).tw,kf,dq. | 72104 |
| 8 | ("copies/ml" or "cells/ml" or "cells per cubic millimeter" or "<=1000copies/mL").tw,kf. | 40351 |
| 9 | ((cells or copies) adj3 (millimeter or mm or ml or milliliter)).tw,kf. | 78384 |
| 10 | (undetectable or (limit? adj2 detect*) or "copies/*").tw,kf. | 253276 |
| 11 | or/6-10 | 431262 |
| 12 | 5 and 11 | 56304 |
| 13 | sexual behavior/ or exp unsafe sex/ | 127746 |
| 14 | ((sex or sexual) adj3 (behavio?r* or unsafe* or partner* or transmi* or condomless or unprotected)).tw,kf,dq. | 84567 |
| 15 | ((seropositiv* or seronegativ* or seroconver* or serodiscordan*) adj4 (partner* or couple*)).tw,kf,dq. | 1923 |
| 16 | 13 or 14 or 15 | 167869 |
| 17 | 12 and 16 | 1765 |
| 18 | (202311* or 2024* or 2025*).dc,dd. | 2376530 |
| 19 | 17 and 18 | 90 |

**EBM Reviews - Cochrane Database of Systematic Reviews [Ovid]**

| EBM Reviews - Cochrane Central Register of Controlled Trials <December 2024>  EBM Reviews - Cochrane Database of Systematic Reviews <2005 to January 8, 2025> | | |
| --- | --- | --- |
| 1 | exp Anti-Retroviral Agents/ | 9511 |
| 2 | ((antiretrovir* or anti-retrovir*) adj (agent? or therapy)).tw. | 6896 |
| 3 | (anti-hiv adj2 (agent? or drug? or therapy)).tw. | 281 |
| 4 | ((CCR5 adj3 (Receptor or Antagonist? or blocker?)) or Atazanavir-Sulfate or Darunavir or Indinavir or Lopinavir or Nelfinavir or Ritonavir or Saquinavir or Raltegravir Potassium or Maraviroc or Enfuvirtide or Cobicistat or Delavirdine or Didanosine or Emtricitabine or Lamivudine or Nevirapine or Rilpivirine or Stavudine or Tenofovir or Trichosanthin or Zalcitabine or Zidovudine).tw. | 10393 |
| 5 | 1 or 2 or 3 or 4 | 17285 |
| 6 | Viral Load/ | 3441 |
| 7 | ((viral or virus) adj2 (load or burden or titer or titre)).tw. | 6743 |
| 8 | ("copies/ml" or "cells/ml" or "cells per cubic millimeter" or "<=1000copies/mL").tw. | 4814 |
| 9 | ((cells or copies) adj3 (millimeter or mm or ml or milliliter)).tw. | 5879 |
| 10 | (undetectable or (limit? adj2 detect*) or "copies/*").tw. | 4818 |
| 11 | or/6-10 | 15360 |
| 12 | 5 and 11 | 6322 |
| 13 | sexual behavior/ or unsafe sex/ | 3161 |
| 14 | Sexual Partners/ | 1062 |
| 15 | ((sex or sexual) adj3 (behavio?r* or unsafe* or partner* or transmi* or condomless or unprotected)).tw. | 5363 |
| 16 | ((seropositiv* or seronegativ* or seroconver* or serodiscordan*) adj4 (partner* or couple*)).tw. | 194 |
| 17 | 13 or 14 or 15 or 16 | 7521 |
| 18 | 12 and 17 | 134 |
| 19 | ("2023" or "2024" or "2025").yr. | 228421 |
| 20 | 18 and 19 | 11 |

**Global Health [Ovid]**

| Global Health <1973 to 2025 Week 03> | | |
| --- | --- | --- |
| 1 | ((antiretrovir* or anti-retrovir*) adj (agent? or therapy)).tw. | 51510 |
| 2 | (anti-hiv adj2 (agent? or drug? or therapy)).tw. | 901 |
| 3 | ((CCR5 adj3 (Receptor or Antagonist? or blocker?)) or Atazanavir-Sulfate or Darunavir or Indinavir or Lopinavir or Nelfinavir or Ritonavir or Saquinavir or Raltegravir Potassium or Maraviroc or Enfuvirtide or Cobicistat or Delavirdine or Didanosine or Emtricitabine or Lamivudine or Nevirapine or Rilpivirine or Stavudine or Tenofovir or Trichosanthin or Zalcitabine or Zidovudine).tw. | 22246 |
| 4 | 1 or 2 or 3 | 64465 |
| 5 | ((viral or virus) adj2 (load or burden or titer or titre)).tw. | 32544 |
| 6 | ("copies/ml" or "cells/ml" or "cells per cubic millimeter" or "<=1000copies/mL").tw. | 14741 |
| 7 | ((cells or copies) adj3 (millimeter or mm or ml or milliliter)).tw. | 17758 |
| 8 | (undetectable or (limit? adj2 detect*) or "copies/*").tw. | 59078 |
| 9 | or/5-8 | 97071 |
| 10 | 4 and 9 | 15670 |
| 11 | ((sex or sexual) adj3 (behavio?r* or unsafe* or partner* or transmi* or condomless or unprotected)).tw. | 45544 |
| 12 | ((seropositiv* or seronegativ* or seroconver* or serodiscordan*) adj4 (partner* or couple*)).tw. | 967 |
| 13 | 11 or 12 | 45933 |
| 14 | 10 and 13 | 666 |
| 15 | ("2023" or "2024" or "2025").yr. | 401727 |
| 16 | 14 and 15 | 61 |

**Scopus [Elsevier]**

| ( TITLE-ABS ( ( ( antiretrovir* OR anti-retrovir* ) W/1 ( agent* OR therapy ) ) OR ( anti-hiv W/2 ( agent* OR drug* OR therapy ) ) OR ( ( ccr5 W/3 ( receptor OR antagonist* OR blocker* ) ) OR atazanavir-sulfate OR darunavir OR indinavir OR lopinavir OR nelfinavir OR ritonavir OR saquinavir OR raltegravir AND potassium OR maraviroc OR enfuvirtide OR cobicistat OR delavirdine OR didanosine OR emtricitabine OR lamivudine OR nevirapine OR rilpivirine OR stavudine OR tenofovir OR trichosanthin OR zalcitabine OR zidovudine ) ) ) AND ( TITLE-ABS ( ( ( viral OR virus ) W/2 ( load OR burden OR titer OR titre ) ) OR ( "copies/ml" OR "cells/ml" OR "cells per cubic millimeter" OR "<=1000copies/mL" ) OR ( ( cells OR copies ) W/3 ( millimeter OR mm OR ml OR milliliter ) ) OR ( undetectable OR ( limit* W/2 detect* ) OR "copies/*" ) ) ) AND ( TITLE-ABS ( ( ( sex OR sexual ) W/3 ( behavio*r* OR unsafe* OR partner* OR transmi* OR condomless OR unprotected ) ) OR ( ( seropositiv* OR seronegativ* OR seroconver* OR serodiscordan* ) W/4 ( partner* OR couple* ) ) ) ) AND PUBYEAR > 2022 | 467+51 |
| --- | --- |

**Appendix 2: PRISMA Abstract checklist**

| **Section and Topic** | **Item #** | **Checklist item** | **Reported (Yes/No)** |
| --- | --- | --- | --- |
| **TITLE** | | |  |
| Title | 1 | Identify the report as a systematic review. | yes |
| **BACKGROUND** | | |  |
| Objectives | 2 | Provide an explicit statement of the main objective(s) or question(s) the review addresses. | yes |
| **METHODS** | | |  |
| Eligibility criteria | 3 | Specify the inclusion and exclusion criteria for the review. | yes |
| Information sources | 4 | Specify the information sources (e.g. databases, registers) used to identify studies and the date when each was last searched. | yes |
| Risk of bias | 5 | Specify the methods used to assess risk of bias in the included studies. | yes |
| Synthesis of results | 6 | Specify the methods used to present and synthesise results. | yes |
| **RESULTS** | | |  |
| Included studies | 7 | Give the total number of included studies and participants and summarise relevant characteristics of studies. | yes |
| Synthesis of results | 8 | Present results for main outcomes, preferably indicating the number of included studies and participants for each.  If meta-analysis was done, report the summary estimate and confidence/credible interval. If comparing groups, indicate the direction of the effect (i.e. which group is favoured). | yes  no |
| **DISCUSSION** | | |  |
| Limitations of evidence | 9 | Provide a brief summary of the limitations of the evidence included in the review (e.g. study risk of bias, inconsistency and imprecision). | yes |
| Interpretation | 10 | Provide a general interpretation of the results and important implications. | yes |
| **OTHER** | | |  |
| Funding | 11 | Specify the primary source of funding for the review. | yes |
| Registration | 12 | Provide the register name and registration number. | yes |

From: Page MJ, McKenzie JE, Bossuyt PM, Boutron I, Hoffmann TC, Mulrow CD, et al. The PRISMA 2020 statement: an updated guideline for reporting systematic reviews. BMJ 2021;372:n71. doi: 10.1136/bmj.n71

**Appendix 3: PRISMA 2020 checklist**

| **Section and Topic** | **Item #** | **Checklist item** | **Page number** |
| --- | --- | --- | --- |
| **TITLE** | | |  |
| Title | 1 | Identify the report as a systematic review. | 1 |
| **ABSTRACT** | | |  |
| Abstract | 2 | See the PRISMA 2020 for Abstracts checklist. | 3 |
| **INTRODUCTION** | | |  |
| Rationale | 3 | Describe the rationale for the review in the context of existing knowledge. | 6 |
| Objectives | 4 | Provide an explicit statement of the objective(s) or question(s) the review addresses. | 6-7 |
| **METHODS** | | |  |
| Eligibility criteria | 5 | Specify the inclusion and exclusion criteria for the review and how studies were grouped for the syntheses. | 6  Appendix 6  Appendix 7 |
| Information sources | 6 | Specify all databases, registers, websites, organisations, reference lists and other sources searched or consulted to identify studies. Specify the date when each source was last searched or consulted. | 8 |
| Search strategy | 7 | Present the full search strategies for all databases, registers and websites, including any filters and limits used. | Appendix 1 |
| Selection process | 8 | Specify the methods used to decide whether a study met the inclusion criteria of the review, including how many reviewers screened each record and each report retrieved, whether they worked independently, and if applicable, details of automation tools used in the process. | 8 |
| Data collection process | 9 | Specify the methods used to collect data from reports, including how many reviewers collected data from each report, whether they worked independently, any processes for obtaining or confirming data from study investigators, and if applicable, details of automation tools used in the process. | 8-10 |
| Data items | 10a | List and define all outcomes for which data were sought. Specify whether all results that were compatible with each outcome domain in each study were sought (e.g. for all measures, time points, analyses), and if not, the methods used to decide which results to collect. | 10  Table 1  Table 3  Figure 2  Table 4 |
|  | 10b | List and define all other variables for which data were sought (e.g. participant and intervention characteristics, funding sources). Describe any assumptions made about any missing or unclear information. | 9-10 |
| Study risk of bias assessment | 11 | Specify the methods used to assess risk of bias in the included studies, including details of the tool(s) used, how many reviewers assessed each study and whether they worked independently, and if applicable, details of automation tools used in the process. | 9-10  Table 2 |
| Effect measures | 12 | Specify for each outcome the effect measure(s) (e.g. risk ratio, mean difference) used in the synthesis or presentation of results. | n/a |
| Synthesis methods | 13a | Describe the processes used to decide which studies were eligible for each synthesis (e.g. tabulating the study intervention characteristics and comparing against the planned groups for each synthesis (item #5)). | 10 |
|  | 13b | Describe any methods required to prepare the data for presentation or synthesis, such as handling of missing summary statistics, or data conversions. | n/a |
|  | 13c | Describe any methods used to tabulate or visually display results of individual studies and syntheses. | n/a |
|  | 13d | Describe any methods used to synthesize results and provide a rationale for the choice(s). If meta-analysis was performed, describe the model(s), method(s) to identify the presence and extent of statistical heterogeneity, and software package(s) used. | n/a  cannot be provided due to insufficient reporting and heterogeneity of studies |
|  | 13e | Describe any methods used to explore possible causes of heterogeneity among study results (e.g. subgroup analysis, meta-regression). | n/a  cannot be provided due to insufficient reporting and heterogeneity of studies |
|  | 13f | Describe any sensitivity analyses conducted to assess robustness of the synthesized results. | n/a  cannot be provided due to insufficient reporting and heterogeneity of studies |
| Reporting bias assessment | 14 | Describe any methods used to assess risk of bias due to missing results in a synthesis (arising from reporting biases). | cannot be provided due to insufficient reporting and heterogeneity of studies |
| Certainty assessment | 15 | Describe any methods used to assess certainty (or confidence) in the body of evidence for an outcome. | 10 |
| **RESULTS** | | |  |
| Study selection | 16a | Describe the results of the search and selection process, from the number of records identified in the search to the number of studies included in the review, ideally using a flow diagram. | 8, 9  Figure 1 |
|  | 16b | Cite studies that might appear to meet the inclusion criteria, but which were excluded, and explain why they were excluded. | 11-13 |
| Study characteristics | 17 | Cite each included study and present its characteristics. | Table 1 |
| Risk of bias in studies | 18 | Present assessments of risk of bias for each included study. | Table 2 |
| Results of individual studies | 19 | For all outcomes, present, for each study: (a) summary statistics for each group (where appropriate) and (b) an effect estimate and its precision (e.g. confidence/credible interval), ideally using structured tables or plots. | (a) Yes, summary of possible HIV transmissions is in Table 2  Items mentioned in (b) cannot be provided due to insufficient reporting and heterogeneity of studies |
| Results of syntheses | 20a | For each synthesis, briefly summarise the characteristics and risk of bias among contributing studies. | Table 2 |
|  | 20b | Present results of all statistical syntheses conducted. If meta-analysis was done, present for each the summary estimate and its precision (e.g. confidence/credible interval) and measures of statistical heterogeneity. If comparing groups, describe the direction of the effect. | n/a  cannot be provided due to insufficient reporting and heterogeneity of studies |
|  | 20c | Present results of all investigations of possible causes of heterogeneity among study results. | 7 |
|  | 20d | Present results of all sensitivity analyses conducted to assess the robustness of the synthesized results. | n/a  cannot be provided due to insufficient reporting and heterogeneity of studies |
| Reporting biases | 21 | Present assessments of risk of bias due to missing results (arising from reporting biases) for each synthesis assessed. | Yes, due to insufficient reporting in studies (pages 13-15) |
| Certainty of evidence | 22 | Present assessments of certainty (or confidence) in the body of evidence for each outcome assessed. | 9, 11-14, Table 2 |
| **DISCUSSION** | | |  |
| Discussion | 23a | Provide a general interpretation of the results in the context of other evidence. | 16-20 |
|  | 23b | Discuss any limitations of the evidence included in the review. | 20 |
|  | 23c | Discuss any limitations of the review processes used. | 20 |
|  | 23d | Discuss implications of the results for practice, policy, and future research. | 19-22 |
| **OTHER INFORMATION** | | |  |
| Registration and protocol | 24a | Provide registration information for the review, including register name and registration number, or state that the review was not registered. | 3 |
|  | 24b | Indicate where the review protocol can be accessed, or state that a protocol was not prepared. | 7 |
|  | 24c | Describe and explain any amendments to information provided at registration or in the protocol. | 7,8 |
| Support | 25 | Describe sources of financial or non-financial support for the review, and the role of the funders or sponsors in the review. | 23 |
| Competing interests | 26 | Declare any competing interests of review authors. | 26 |

**Appendix 4: Synthesis Without Meta-analysis (SWiM) checklist**

| **SWiM** **is** **intended** **to** **complement** **and** **be** **used** **as** **an** **extension** **to** **PRISMA** | | | | |
| --- | --- | --- | --- | --- |
| **SWiM** **reporting**  **item** | **Item** **description** | **Page** **in** **manuscript**  **where** **item** **is** **reported** | | **Other*** |
| *Methods* | | | | |
| **1** Grouping studies for synthesis | 1a) Provide a description of, and rationale for, the groups used in the synthesis (e.g., groupings of populations, interventions, outcomes, study design) | 10, 11, Table 1 | |  |
|  | 1b) Detail and provide rationale for any changes made subsequent to the protocol in the groups used  in the synthesis | 7, 8 | |  |
| **2** Describe the standardized metric and transformation methods used | Describe the standardized metric for each outcome. Explain why the metric(s) was chosen, and describe any methods used to transform the intervention effects, as reported in the study, to the standardized metric, citing any methodological guidance consulted | 11-13 | |  |
| **3** Describe the  synthesis methods | Describe and justify the methods used to synthesize the effects for each outcome when it was not possible to undertake a meta-analysis of effect estimates | n/a | |  |
| **4** Criteria used to prioritize results for summary and synthesis | Where applicable, provide the criteria used, with supporting justification, to select the particular studies, or a particular study, for the main synthesis or to draw conclusions from the synthesis (e.g., based on study design, risk of bias assessments, directness in relation to the review question) | 10-15 | |  |
| **5** Investigation of heterogeneity in reported effects | State the method(s) used to examine heterogeneity in reported effects when it was not possible to undertake a meta-analysis of effect estimates and its extensions to investigate heterogeneity | 7, 9-10 |  | |
| **6** Certainty of evidence | Describe the methods used to assess certainty of the synthesis findings | 9-10, Table 2 |  | |
| **7** Data presentation methods | Describe the graphical and tabular methods used to present the effects (e.g., tables, forest plots, harvest plots).  Specify key study characteristics (e.g., study design, risk of bias) used to order the studies, in the text and any tables or graphs, clearly referencing the studies included | n/a  Table 2 |  | |
| *Results* | | | | |
| **8** Reporting results | For each comparison and outcome, provide a description of the synthesized findings, and the certainty of the findings. Describe the result in language that is consistent with the question the synthesis addresses, and indicate which studies contribute to the synthesis | Table 3  Figure 2  Table 4 |  | |
| *Discussion* |  |  |  | |
| **9** Limitations of the synthesis | Report the limitations of the synthesis methods used and/or the groupings used in the synthesis, and how these affect the conclusions that can be drawn in relation to the original review question | 20 |  | |

PRISMA=Preferred Reporting Items for Systematic Reviews and Meta-Analyses.

*If the information is not provided in the systematic review, give details of where this information is available (e.g., protocol, other published papers (provide citation details), or website (provide the URL)).

**Appendix 5. Ratings of risk of Bias using QUIPS tool**

| Outcome | Study | **Study participation** | **Study attrition** | **Prognostic factor measurement** | **Outcome measurement** | **Study confounding** | **Statistical Analysis and reporting** | **Overall risk of bias** |
| --- | --- | --- | --- | --- | --- | --- | --- | --- |
| HIV transmission | Bavinton et al., 2018 |  |  |  |  |  |  |  |
|  | Rodger et al., 2019 |  |  |  |  |  |  |  |
|  | Rodger et al., 2016 |  |  |  |  |  |  |  |
|  | Sun et al., 2020 |  |  |  |  |  |  |  |
|  | Mujugira et al., 2016 |  |  |  |  |  |  |  |
|  | Eshleman et al., 2017 |  |  |  |  |  |  |  |
|  | Birungi et al., 2015 |  |  |  |  |  |  |  |
|  | Donnell et al., 2010 |  |  |  |  |  |  |  |
|  | Han et al. 2024 |  |  |  |  |  |  |  |

Green= low risk of bias; yellow= moderate risk of bias; red= high risk of bias. High risk of bias was due to confounding. Frequent condom use was the most impactful confounding variable driving the high risk of bias.

**Appendix 6. Rationale for Inclusion of Studies, Strengths, and Limitations**

| Article | Reason for Inclusion |
| --- | --- |
| Donnell 2010 | Not included in last review  Within viral load threshold  ART initiation close to time of transmission  Only about 7% of participants had unprotected sex  Paper is older and data is old |
| Birungi 2015 | Not included in last review  Within viral load threshold of interest  High condom use  PY for couples with infections by VL but not for couples with no infection |
| Rodgers 2016 | Very clearly reported. Used in last review  <200 copies/mL |
| Mujugira 2017 | Not included in the last review  Phylogenetic analysis and VL testing at time of transmission completed  3 infections in 168 PY during the first six months of ART use, 1 below 1000 copies/mL |
| Eshleman 2017 | Not included in last review  Reported transmissions <400 copies/mL  only about 5% of participants had unprotected sex  Initiation of ART too close to time of transmission (Earliest possible transmission may have occurred before ART initiation or shortly after) |
| Bavinton 2018 | Very clearly reported. Used in last review  <200 copies/mL |
| Rodgers 2019 | Very clearly reported. Used in last review  <200 copies/mL |
| Sun 2020 | Not included in last review  All participants <50 copies/ mL for at least 6 months  At minimum 142 x 0.5 =71 person years, which is low |
| Han 2024 | Not included in last review  Within viral load threshold  Initiation of ART close to time of transmission |

VL, viral load; PY, person years; ART, antiretroviral therapy.

**Appendix 7. Rationale for Articles Excluded**

| **Article** | **Reason for Exclusion** |
| --- | --- |
| Melo 2008 | Very small sample size of 41 serodiscordant couples  No phylogenetic analysis performed |
| Apondi 2011 | Not included in the last review  High condom use ~58% in serodiscordant couples  Low sample size of 62 couples  The viral load associated with transmission is unclear |
| He 2013 | High risk of confounding  Only small percentage of participants engaged in non-consistent condom use  VL measurements done only at baseline and F/U.  Not sure how close F/U VL measurements were to time of infection  Great span of time (12 months) between VL and transmission. |
| Del Romero 2014 | 6/32 couples engaged in unprotected sex, the rest used condom prophylaxis  Data from study is prior to 2005  No phylogenetic analysis performed |
| Yang 2015 | Not included in the last review  <1000 copies/mL  5 transmissions reported >1000 copies/mL but no phylogenetic analysis was done |
| Melo 2019 | Moderate risk of bias  No phylogenetic analysis performed between heterosexual couples, but authors insist on no outside relationship as reported by the couples  Adherence to ART was self-reported (social desirability bias)  36% of seroconversions were seen in participants engaging in unprotected sex, the others reported condom usage  Unclear the exact VL that corresponded to each couple |
| He 2024 | No individual PLHIV VL measurements reported  No detailed information provided on individual transmissions |
| Mathews 2024 | Two HIV negative clients seroconverted, however, no detailed information provided  No individual PLHIV VL measurements reported  No phylogenetic test or linkage of infection to sexual partner |
| Munishi 2023 | Wrong study design - Umbrella review |

VL, viral load; PY, person years; ART, antiretroviral therapy.
